# Treating symptomatic hyperprolactinemia in women with schizophrenia: presentation of the ongoing DAAMSEL clinical trial (Dopamine partial Agonist, Aripiprazole, for the Management of Symptomatic ELevated prolactin)

**DOI:** 10.1186/1471-244X-13-214

**Published:** 2013-08-22

**Authors:** Deanna L Kelly, Heidi J Wehring, Amber K Earl, Kelli M Sullivan, Faith B Dickerson, Stephanie Feldman, Robert P McMahon, Robert W Buchanan, Dale Warfel, William R Keller, Bernard A Fischer, Joo-Cheol Shim

**Affiliations:** 1 School of Medicine, Maryland Psychiatric Research Center, University of Maryland Baltimore, Baltimore, Maryland, USA; 2 Sheppard and Enoch Pratt Hospital, Baltimore, Maryland, USA; 3 VA Capital Network (VISN 5) Mental Illness Research, Education, and Clinical Center (MIRECC), Baltimore, Maryland, USA; 4 Department of Psychiatry and Clinical Trial Center, Busan Paik Hospital, Inje University, Busan, South Korea

**Keywords:** Women, Prolactin, Amenorrhea, Galactorrhea, Clinical trial, Sexual dysfunction, Osteoporosis, Aripiprazole

## Abstract

Prolactin elevations occur in people treated with antipsychotic medications and are often much higher in women than in men. Hyperprolactinemia is known to cause amenorrhea, oligomenorrhea, galactorrhea and gynecomastia in females and is also associated with sexual dysfunction and bone loss. These side effects increase risk of antipsychotic nonadherence and suicide and pose significant problems in the long term management of women with schizophrenia. In this manuscript, we review the literature on prolactin; its physiology, plasma levels, side effects and strategies for treatment. We also present the rationale and protocol for an ongoing clinical trial to treat symptomatic hyperprolactinemia in premenopausal women with schizophrenia. More attention and focus are needed to address these significant side effects and help the field better personalize the treatment of women with schizophrenia.

## Background

Hyperprolactinemia and related consequences are known side effects of several first and second generation antipsychotics. Elevation of serum prolactin by antipsychotic drug and clinical manifestations associated with this condition has been recognized for more than three decades [1]. Nevertheless, the syndrome of antipsychotic-induced hyperprolactinemia has been relatively neglected. A review article in 2004 reported that from 1966–2004 a search of the Medline database returned 1717 journal articles on antipsychotics and extrapyramidal side effects, yet only 375 articles on antipsychotics and hyperprolactinemia [2]. Hallewell and colleagues [3] performed a survey and reported that both psychiatrists and psychiatric nurses underestimate the prevalence of key symptoms associated with hyperprolactinemia. A recent cohort study reported that among 194 schizophrenia and bipolar patients receiving antipsychotics from a single community mental health facility in the UK, 38% had prolactin levels above the upper limit of normal, two thirds of whom had significantly elevated levels with clinical consequences. Women were particularly at risk for elevations and associated consequences, with over 50% of women in the study having abnormal prolactin levels [4]. In recent years this issue has begun to gain more attention. The last iteration of the Patient Outcomes Research Team (PORT) guidelines specifically addresses the issue of hyperprolactinemia associated with antipsychotic use however, too few randomized double blind trials were available to make and evidence based recommendation [5]. Also very recently, suicide risk has been found to be significantly increased with symptomatic hyperprolactinemia [6]. Our group has been addressing hyperprolactinemia for over a decade and are currently running an adjunct treatment trial to improve outcomes in women with this distressing side effect. In this manuscript, we review the physiology and treatment of elevated prolactin secondary to antipsychotic medications and present the rationale and protocol for an ongoing clinical trial that addresses this issue in women.

### Prolactin physiology

The main physiologic function of prolactin is to cause breast enlargement during pregnancy and milk production during lactation. Prolactin is an amino acid polypeptide hormone secreted by the lactotroph cells of the anterior pituitary. In a pulsatile manner, this 199 amino acid hormone is released with 13–14 peaks per day and an interpulse interval of about 95 minutes. Prolactin levels show a circadian rhythm, with a maximum level reached about 4 hours after the onset of sleep and a minimum level reached about 6 hours after waking [7]. Transient and mild increases in prolactin levels occur in response to sexual activity, stress and eating [8]. In women, prolactin levels are typically higher during mid cycle and the second half of the menstrual cycle. During pregnancy, serum prolactin levels increase to reach levels 10–20 times the normal value. Prolactin elevations during and post pregnancy can reach 200 ng/ml at full term and 300 ng/ml while nursing [9]. Levels generally normalize approximately 3 weeks postpartum in the absence of nursing. The reductions in sexual functioning and fertility associated with nursing may have evolutionary advantages. In animals, prolactin is known to have an important function in promoting maternal behavior; however, this has not been proven in humans. Because women have a physiologic purpose for prolactin elevations, the female prolactin system may be more likely to respond to stimuli, which may underlie significantly higher prolactin levels in women versus men after dopamine antagonist treatment. Men are more likely to have a buffer system in place to guard against significant prolactin elevations.

### Normal prolactin levels

Despite these variations in serum prolactin levels, both within and between individuals, there is a reasonable consensus regarding the upper limit of the normal range. Some authorities have suggested that the upper limit is 25 ng/ml in both men and women [10,11]. Others have used >10 ng/ml in males and >20 in females [12] and yet others have used >15-16 ng/ml in both sexes [13,14]. The most recent and more conservative reports suggest that >18-20 ng/ml in men and >24 ng/ml in non-pregnant, non-nursing women should be considered the upper limits for prolactin serum concentrations [15-18].

### Prolactin control

#### Inhibition and stimulation

Prolactin secretion is under the control of various peptide and steroid hormones and neurotransmitters [19]. Dopamine, however, is the predominant prolactin-inhibiting factor in humans and animals. In the tuberoinfundibular neurons of the hypothalamus, dopamine is produced and released from nerve endings. It is transported by the portal hypophyseal circulation to the pituitary, where it binds to dopamine D2 receptors on the membrane of the lactotroph cells [20]. The presence of dopamine suppresses the release of prolactin to a minimum. Dopamine D2 antagonists, such as most antipsychotics, bind to the D2 receptors and lead to the release of prolactin in the lactotroph cells. Animal and human studies demonstrate that serotonin may stimulate prolactin secretion [21] as well as several peptide neurotransmitters, including thyrotropin releasing hormone and cholecystokinin [19]. These effects are not as well characterized or robust as that of dopamine on prolactin control.

### Antipsychotic medications and prolactin

Virtually all antipsychotic drugs have the capacity to block D2 receptors in the mesolimbic and mesocortical areas, which is believed to be integral for efficacy of these agents. In other areas of the brain, however, areas of D2 blockade can cause significant adverse effects. Extrapyramidal side effects such as Parkinsonism result from D2 antagonism in the striatum, while blockade of D2 receptors on lactotroph cells causes hyperprolactinemia, since the main inhibitory influence (i.e., dopamine) is removed. Conventional antipsychotics in people with schizophrenia as well as healthy individuals cause prolactin elevations within hours of taking these agents [22,23]. Studies with first generation antipsychotics (FGAs) (3–9 weeks) demonstrate that therapeutic doses of any of these medications cause prolactin elevations of up to 10 fold and elevations have been reported to correlate to the dose [24]. The propensity to elevate prolactin levels varies among second-generation antipsychotics (SGAs). Clozapine has a low propensity to block dopamine in the tuberinfundibular pathway and has a negligible effect on plasma prolactin levels [25]. Olanzapine causes transient elevations in plasma prolactin levels. During treatment in adults, prolactin levels remain slightly elevated in about 1/3 of patients [26]. As with FGAs, elevation of prolactin with olanzapine appears to be a dose related phenomenon [27]. Mean prolactin levels during 10–30 mg daily treatment with olanzapine are approximately 17 ng/ml, which is higher than that of normals, drug-free patients and clozapine-treated patients [28]. Quetiapine has negligible effects on the elevation of prolactin. In all of the large trials of quetiapine, prolactin levels were reported to decrease from baseline to endpoint during quetiapine treatment and no differences were noted between quetiapine and placebo [29-32]. It appears as though elevations may occur with ziprasidone. In a double-blind study elevated prolactin levels were seen in over 40% of patient treated with ziprasidone at the end of 8 weeks as were over 60% in the olanzapine treated group [33].

Of all the SGAs, risperidone and paliperidone [34] have the highest propensity to elevate plasma prolactin levels, and do so in a dose-related fashion. It is also well established that, while high potency FGAs have prolactin raising capabilities, risperidone and paliperidone elevate prolactin significantly more than FGAs. In a naturalistic cross sectional study in women, prevalence of elevated prolactin (>24 ng/ml) was higher in those prescribed risperidone (88%) as compared to a conventional antipsychotic (48%) [17]. Also, in a double blind 12 week randomized trial by our group, prolactin levels were 50.6 ± 40.4 mg/dl, 24.4 ± 18.5 mg/dl, and 8.2 ± 4.4 mg/dl for risperidone, fluphenazine and quetiapine, respectively (F = 7.5,df = 2, p = 0.005, controlling for sex). The women in this study treated with a fixed risperidone dose of 4 mg/day had mean prolactin levels of 92 ng/ml after 12 weeks of treatment on risperidone, an elevation with potential clinical significance [35].

Aripiprazole treatment has been associated with significant decreases in serum prolactin levels. In a recent double blind randomized trial by Kane and colleagues [36] mean prolactin levels decreased in the aripiprazole group from 33.4 ng/ml to 5.2 ng/ml in the 6 week trial, while prolactin levels in the perphenazine group remained unchanged at 35.8 ng/ml to 35.5 ng/ml. Another recent 4 week double-blind randomized trial reported that prolactin levels were significantly decreased in the aripiprazole group (−9.0 ng/ml) compared to a mean increase of 55.4 ng/ml in the risperidone group. In this study, the percentage of schizophrenia patients who had an abnormal prolactin levels (>25 ng/ml) at endpoint was significantly higher in the risperidone group (93%) than in the aripiprazole group (5%) [37]. The absence of significant prolactin elevation with aripiprazole may be explained by aripiprazole’s partial agonism at D_2_ receptors, in contrast to the D_2_ antagonism of other second-generation agents. Aripiprazole has been shown to act as an agonist in pituitary cells at the molecular level and thus, lactotroph cells do not become blocked by aripiprazole treatment [38].

### Consequences of elevated prolactin

Methodological problems in the existing literature make it difficult to determine the prevalence of symptomatic hyperprolactinemia in persons treated with antipsychotics. Many previous studies have only examined prolactin levels and not assessed its symptomatic consequences. Due to inter-individual differences in responses to prolactin, elevated prolactin may at times be asymptomatic. Elevated prolactin levels with no apparent consequences are more common in males, as women tend to have menstrual period abnormalities and other effects with significantly elevated prolactin levels. Many studies in the past have done a poor job at assessing presence of symptomatic side effects in women. Additionally, cross sectional studies can only show an association between symptoms and raised prolactin; proving a causal relationship can be more complex. Ideally, it requires a follow up design to determine whether reversal of hyperprolactinemia is accompanied by symptom resolution. Few studies have taken these issues into account.

Health professionals often fail to detect antipsychotic induced hyperprolactinemia symptoms [34]. There are several reasons for this, including the nature of the symptoms and the attitudes of both patients and health professionals. Some patients may be embarrassed to discuss endocrine and sexual symptoms, and do not normally volunteer symptoms to the clinical or research staff. Unlike some antipsychotic adverse effects (e.g., extrapyramidal symptoms and weight gain), some symptoms of hyperprolactinemia may not be not visibly stigmatizing, which reduces their detection. Nonadherence to antipsychotic treatment is common with prolactin related side effects and may be due to many of symptoms discussed below. In a large nationwide survey in almost 900 people with schizophrenia taking antipsychotic medications, almost 60% reported nonadherence, with prolactin and endocrine related effects being one of the commonly cited reasons, decreasing the odds of adherence by about 30% [39].

#### a. Galactorrhea

Galactorrhea secondary to prolactin elevation is more common in women than in men. Estimates of the prevalence of galactorrhea vary from 10-50%. A well-conducted study found that 28/150 women (19%) developed galactorrhea within 75 days of commencing conventional antipsychotic treatment [40]. In this study, only 8 women reported the symptom on their own to the clinician, illustrating that symptomatic hyperprolactinemia is often unrecognized or under-reported. Lactation from the breast in the nonpregnant state is most likely distressing to women who suffer from a reality distortion illness and likely also to interfere with social and intimate relationships [41]. Gynecomastia also is common and can occur in both men and women.

#### b. Menstrual abnormalities

Menstrual abnormalities are a frequently occurring side effect that often goes unnoticed and under recognized in clinical trials. Typically, this information is not volunteered and clinical investigators do not evaluate this side effect. Seven cross sectional studies have investigated menstrual irregularities in women with schizophrenia receiving long-term treatment with conventional antipsychotics or risperidone. In these studies the reported prevalences were 25%, 26%, 40%, 45%, 55%, 56% and 78% [12,24,42-46]. While these studies had small sample sizes and no clear definition of oligomenorrhea or amenorrhea, it is still evident that many women may experience these side effects. In a cross sectional study of 42 women prescribed risperidone, 48% experienced abnormal menstrual cycles. In our previous 12-week double blind pilot trial, 100% of female subjects in the risperidone group experienced amenorrhea during treatment [35]. Women who experience amenorrhea or oligomenorrhea are likely infertile or subfertile. Long-term amenorrhea also increases the risk of bone mineral density loss and cardiovascular disease [47].

#### c. Sexual dysfunction

Sexual dysfunction has been implicated as one of the major factors contributing to noncompliance with antipsychotic medications [48-51]. A survey identified “personal relationships” as one of the treatment areas with the most unmet needs noted by people with schizophrenia [52]. Quality of life may be improved with better hormonal and sexual functioning as well [53,54]. It can be hypothesized that a better focus on sexuality and preventing sexual dysfunction in schizophrenia would be a major benefit for improving treatment compliance and life quality.

While sexual dysfunction and diminished sexual functioning during antipsychotic treatment also may be caused by sedation, weight gain, extrapyramidal side effects, tardive dyskinesia, cholinergic antagonism, alpha-adrenergic blockade and calcium channel blockade [55-58], the evidence that elevated prolactin contributes to sexual dysfunction is convincing. Hyperprolactinemia is known to cause hypogonadism and decrease testosterone levels [59]. Prolactin itself appears to have a direct effect on sexual activity as normalization of prolactin levels with bromocriptine has been shown to restore sexual functioning before testosterone levels increase [39]. In non-psychiatric subjects, Faiman [60] stated that 80% of men with serum prolactin levels >50 ng/ml complained of impaired libido and impotence. Ghadirian et al. [45] found that elevated serum prolactin correlated with worse sexual dysfunction in men. Similar results were reported by Burke et al. in the mid 1990s [61]. Other studies have had variable results for correlational type analyses but most generally report higher rates of sexual dysfunction and menstrual disturbances with higher prolactin levels [62-65].

The psychiatric literature contains several reports describing sexual disturbances in schizophrenia, but very few well-designed studies address this issue. Few reports describe sexual function or dysfunction in women. The definition and measurement of sexual dysfunction has been inconsistent, but a few general classifications appear in the existing literature [66]. In men, it appears that the most commonly reported (30-60%) sexual side effects associated with traditional antipsychotics are erection and ejaculation disturbances [67]. Difficulty achieving and maintaining erection is a common complaint as is delayed or inhibited ejaculation, retrograde and spontaneous ejaculation. Diminished libido and decreased orgasm quality are also commonly reported in men. Priapism, a sustained painful erection that can result in permanent impotence, has also been reported [68,69]. Women report decreased libido and orgasmic dysfunction, including difficulty achieving orgasm, changes in the quality of orgasm and anorgasmia [40,59]. Women may also experience dysparenuria secondary to vaginal atrophy and dryness.

Sexual dysfunction appears to be a particularly salient issue in women treated with antipsychotic medications: Howes et al. [14] found an odds ratio (OR) of 15.2 for risk of sexual dysfunction in women with schizophrenia compared to normal controls, compared to an OR 3.7 in men. Recent reports suggest 59% [70] to 80% [71] of women with schizophrenia experience sexual dysfunction, particularly those treated with conventional antipsychotics or risperidone.

#### d. Other adverse effects

##### Bone mineral density

Osteoporosis is a systemic skeletal disease characterized by low bone mass and microarchitectural deterioration of bone tissue, with a consequent increase in bone fragility and susceptibility to fracture. People with schizophrenia represent a high-risk group for developing osteoporosis because of the disease itself, long-term antipsychotic drug treatment, lack of exercise, poor nutrition and high rates of smoking [72,73]. Hyperprolactinemia is known to increase the risk of bone mineral density (BMD) loss in patients with schizophrenia with long term antipsychotic drug treatment, either through secondary hypogonadism [74] or hyperprolactinemia itself [75]. Previous studies have demonstrated that between 32-65% of patients treated with antipsychotic drugs suffer from bone mineral loss, leading to osteoporosis [74,76]. Bone fractures occur more frequently in people with schizophrenia taking antipsychotics than in the nonpsychiatric population [77,78]. In a recent study by our group in Asian patients with schizophrenia, 18/21 (86%) of women with schizophrenia treated with haloperidol had significant bone mineral density loss as compared to 9/23 (39%) of women in the normal control group [15]. Other recent evidence suggests that having amenorrhea or using prolactin elevating antipsychotic drugs are associated with a higher risk for osteoporosis and fracture [79-82]. Very recently, Graham and others [83] published a literature review showing that first generation antipsychotics and certain second generation agents such as risperidone show high prolactin elevations and are associated with an increased risk of osteoporosis and fracture.

##### Pituitary tumors

Lactotroph cells normally occupy 10-15% of the pituitary gland. Knocking out the Dc receptor in mice results in a striking progressive increase in lactotroph cells in the pituitary gland and the rate of pituitary tumors [84]. In these same mice, prolactin levels are 6 times higher in females as compared to males and females develop tumors much earlier than males. Long term treatment with antipsychotics in mice have led to the development of pituitary adenomas and adenocarcinomas [85]. Recent pharmacovigilance studies in humans have found through the FDA’s Adverse Event Reporting System that a significantly greater proportion of pituitary tumors were reported with risperidone than with other available antipsychotics [85,86]. Another prospective study found that pituitary volume significantly increases over one year in people treated with prolactin elevating antipsychotics [87]. The true risk of pituitary tumor with prolactin elevating medications remains unclear. Among the few studies that have addressed whether dopamine receptor antagonists increase risk of breast cancer, results have been conflicting [88]. However, until more data is available, it remains of concern with long term use.

##### Suicide

A major impetus for this review and presentation of our clinical trial protocol was a BMC Psychiatry paper published in June of 2012 that examined risk factors for suicidal behaviors in a large 3 year multinational study (SOHO study) with over 8,000 adult patients with schizophrenia. This was the first study to examine prolactin related effects as a risk factor of suicide. The authors report that prolactin related effects were profoundly associated with increased suicide risk: amenorrhea increased the suicide risk by 64%, galactorrhea by 163% and gynecomastia by 200%. They also found that 84% of the patients with prolactin related adverse effects were female [6]. The exact mechanism for the suicide risk increase is unclear. Potential causes include nonadherence to treatment, low quality of life associated with sexual dysfunction or increased rates of depression and anxiety associated with prolactin elevation effects [89-91]. Nonetheless, our increasing knowledge of significant prolactin related side effects and this new data on very large relative increases in risks of suicidality associated with these side effects mandate increased attention to prolactin-mediated adverse effects of antipsychotic treatments and a search for effective treatments for antipsychotic-induced hyperprolactinemia, particularly in women.

Our group has been working on identification [18], effective rating [35,57,92], consequences [15,35], recognition [57,68,93] and effective treatment [35,94] of prolactin-associated side effects for over 13 years. We have identified what we think to be the most effective strategy for managing these effects in women and are now running an NIMH funded R01 double blind, placebo controlled randomized clinical trial to address high prolactin levels with associated symptoms in women with schizophrenia, the DAAMSEL study (Dopamine Partial Agonist, Aripiprazole, for the Management of Symptomatic ELevated Prolactin) It is a 4 year study with a target enrollment of 50 women with schizophrenia treated with risperidone, paliperidone or FGAs and with elevated prolactin and sexual side effects (menstrual abnormalities or galactorrhea) at baseline. Participants will be followed for 16 weeks on adjunct aripiprazole or placebo. Outcome measures include resolution of menstrual abnormalities or galactorrhea, improvement in sexual functioning, prolactin level improvement, changes in bone resorption measures, psychiatric symptoms, quality of life and related metabolic effects. We are pleased to bring attention to our ongoing study and to publish protocol details as ongoing attention to clinical trials will help improve recognition and reporting of side effects related to antipsychotic-induced prolactin elevations as well as impact on study recruitment (NCT#01338298). Below is the rationale for why we use low dose aripiprazole and a summary of the study methodology.

### Treatment rationale

Few evidence-based treatment options are available for hyperprolactinemia and associated side effects in patients who are treated with a potent D2 blocking drug. A reduction in dose in the offending antipsychotic is the simplest treatment strategy, but its effectiveness is questionable and it carries the risk or precipitating an exacerbation or relapse of psychotic symptoms.

Switching to a prolactin sparing antipsychotic may be effective for decreasing prolactin [16,95-100]. However, these studies provide insufficient evidence to examine the effects of switching on symptom changes and relapse rates or on symptomatic improvement of prolactin related side effects and the risk of relapse is present [95,101]. Lee et al. [96] reported that while all patients switched from risperidone to aripiprazole have amelioration of symptomatic hyperprolactinemia, 29% of the patients experienced aggravated auditory hallucinations. We also have pilot data in 40 women previously psychiatric stable on risperidone but switched to aripiprazole due to prolactin-related symptoms; of these 40% (16/40) had exacerbations in psychotic symptoms. Kuloglu et al. [102] did have 5 women switch successfully, however, the team warn against the accumulating literature suggesting increases in psychotic symptoms during and after a taper or switch to aripiprazole monotherapy. Clinicians do not appear to have as much confidence in the efficacy of monotherapy with aripiprazole compared to risperidone, as evidenced by the smaller market share of aripiprazole relative to other first line antipsychotics. No data are available from large-scale non-industry sponsored clinical trials comparing the efficacy of aripiprazole to the efficacy with risperidone or other second generation antipsychotics. Observational studies have reported greater effectiveness for symptom improvement with olanzapine and risperidone than with aripiprazole [103,104].

Other pharmacologic treatments for prolactin elevation have been tried but have side effects of their own. Bromocriptine, a dopamine agonist has been investigated in several studies for its potential to decrease prolactin levels. Yuan et al. [105] studied the effects of bromocriptine (5 mg) compared to an herbal preparation, Peony-Glycyrrhiza Decoction (PGD) in 20 women with elevated and symptomatic hyperprolactinemia. In both groups, prolactin levels were reduced in about one quarter of subjects; however, of those with improvements in prolactin, more PGD subjects had a resolution of prolactin side effects (56% vs. 21-38%). Another group studied the effect of bromocriptine compared to placebo for drug induced hyperprolactinemia in an 8 week randomized single blind study. Six of 45 (13%) resumed menstruation on bromocriptine compared to 1/15 (7%) on placebo. A 2.5 mg dose of bromocriptine was not effective while 4/15 (27%) treated with 10 mg/day regained their menstrual period [106]. However, dopamine agonists such as bromocriptine have also been associated with worsening of psychosis [107].

In an open label study testing the effectiveness of cabergoline, an ergot derivative and full dopamine agonist, to improve prolactin related side effects, 11/19 (58%) male and female participants with schizophrenia had improvement in their symptoms [108]. However, cardiopulmonary complications are a concern with ergot derived dopamine agonists [109]. Metformin was recently studied as an adjunct to antipsychotic treatment in a randomized double blind placebo controlled trial. Sixty seven percent of those assigned to metformin resumed their menstruation compared to 5% assigned to placebo in a six month period [110]. In other studies, selegiline and cyproheptadine were not effective for sexual dysfunction and one small double blind trial has found benefits with sildenafil for sexual dysfunction but this was not focused on prolactin as a culprit [111,112].

As mentioned, our preliminary work has shown that aripiprazole can significantly improve hyperprolactinemia and related side effects as an adjunct to haloperidol in people with schizophrenia [94]. We have also found that aripiprazole added to dopamine raising antipsychotics can decrease prolactin levels and lead to women regaining their menstrual periods. This strategy is highly effective at decreasing prolactin levels and improving related side effects.

**Figure 1:**
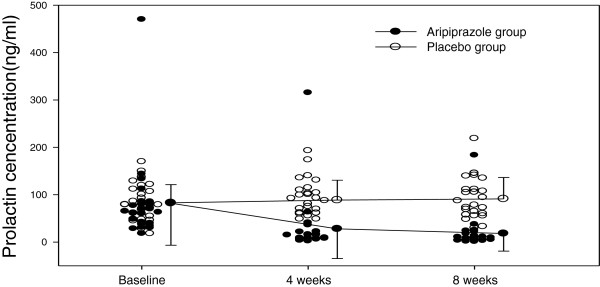
Change in Prolactin Levels over 8 weeks in a Double Blind Trial: Aripiprazole vs. Placebo.

There are a few case reports with aripiprazole improving hyperprolactinemia in patients as compared to their previous treatments [113-115]. Aripiprazole monotherapy [116-118] in double blind trials has shown improvement in prolactin levels compared to baseline. Kane et al. [119] completed a large multicenter 16 double blind placebo controlled study of aripiprazole (2–15 mg/day) in addition to a stable regiment of quetiapine or risperidone. While there were no statistically significant decreases in clinical psychiatric symptoms, aripiprazole was found to improve prolactin elevations (in both men and women), with the authors suggesting this deserves further study as a treatment. In a published study by our group in both men and women we found that adjunct aripiprazole therapy with haloperidol significantly decreased prolactin in the majority of patients relative to placebo [94]. Figure 1 below demonstrates the significant effect of adjunct aripiprazole in patients with schizophrenia having elevated prolactin at baseline (N = 56).

We also have pilot data to suggest that up to 15 mg of aripiprazole can resolve elevated prolactin in women with amenorrhea. Twenty-one of 24 (88%) of women in an open label study of adjunct aripiprazole experienced resolution of their menstrual periods by 8–16 weeks, as well as significant decreasing (from 96.6 ng/ml to 28.1 ng/ml means) their prolactin levels [120]. Yasui-Furukori [121] et al. also found significant improvements in prolactin and symptoms with 4 weeks of adjunct aripiprazole (3,6,9 and 12 mg) with the most robust improvements seen at the 9 and 12 mg/day dose.

In addition to the prolactin improvements with adjunct aripiprazole, there is also data to suggest that aripiprazole augmentation to existing antipsychotics may help improve residual psychiatric symptoms (positive, negative, depressive symptoms). Aripiprazole as an adjunctive treatment has been most studied in combination with clozapine treatment in schizophrenia [122-130]. Adjunct aripiprazole has also been shown in double blind trials to reduce metabolic side effects of olanzapine [131,132] and clozapine [132].

In a review of 31 studies of strategies to treat hyperprolactinemia, Nunes et al. [111] found that only aripiprazole appears to have efficacy. Thus, most data suggests that aripriprazole10-30 mg/day is effective at decreasing prolactin, improving prolactin related side effects and possibly improving residual psychiatric symptoms. The 15 mg/day dose, lowest of the recommended range, appears to offer the greatest tolerability while providing efficacy.

It is important to note that in addition to pharmacologic treatment strategies such as low dose aripiprazole, patients should be made aware of these potential prolactin-related side effects and periodic checks should be made to monitor and evaluate them [133]. Several investigators and clinicians in the field have published recently on the importance of recognition and treatment and that we can possibly halt or potentially even reverse associated bone loss [134].

## Methods/Design

### Aims

The primary study aim of this recently started IRB approved study is to determine if adjunct aripiprazole will resolve or improve prolactin related hormonal side effects (amenorrhea, oligomenorrhea, galactorrhea). We hypothesize as our primary outcome that adjunct aripiprazole will resolve hormonal effects in women with schizophrenia or schizoaffective disorder and symptomatic hyperprolactinemia stabilized on risperidone, paliperidone, or FGAs. Additionally, we have four secondary aims. First, to test whether adjunctive aripiprazole will improve psychiatric symptoms (depression/anxiety, negative symptoms, positive symptoms), perceived quality of life, sexual functioning and perceived wellness. We hypothesize that aripiprazole will improve psychiatric symptoms, quality of life, sexual functioning and perceived wellness relative to placebo in women stabilized on risperidone, paliperidone, or FGAs. Second, to identify if adjunct aripiprazole will improve bone turnover as measured by assays of osteoblastic and osteoclastic activity. We hypothesize that osteoblastic activity will be greater and osteoclastic activity will be less in women treated with adjunct aripiprazole relative to placebo in women stabilized on risperidone, paliperidone, or FGAs. Third, to examine side effects of adjunct aripiprazole vs. placebo. We hypothesize that side effect rates will be similar in the two groups, with the exception of improved metabolic (weight, lipids, glucose) and prolactin-related side effects on adjunct aripiprazole. Our fourth and final secondary aim is to evaluate the mediator effects of estrogen, progesterone and prolactin on quality of life, bone turnover and sexual functioning. In exploratory analyses, we will examine the relationship of sex hormone changes to various outcomes. Sex hormone levels may predict improvement and serve as a biomarker for predicting outcome or bone turnover and determining optimal aripiprazole dose for individual patients. For example, women who have return of menses but do not ovulate, as evidenced by lack of luteal progesterone surge, may have less improvement in bone turnover and sexual side effects.

### Overview

Our ongoing study is a 16-week, double blind, placebo controlled randomized trial of aripiprazole (N = 25) or placebo (N = 25) added to an existing stabilized regimen of prolactin elevating antipsychotics (risperidone, paliperidone, haloperidol, fluphenazine, perphenazine, loxapine). Subjects are seen every two weeks during the study. At baseline all patients receive a full medical and psychiatric evaluation to ensure there are no medical concerns. All subjects must sign informed consent and pass a quiz, the Evaluation of Signed Consent (ESC).

#### Inclusion and exclusion criteria

##### Inclusion criteria

1. Subjects will be females of any race, with an age range of 18-50.

2. Subjects will meet DSM-IV TR [135] criteria for either schizophrenia or schizoaffective disorder. A best estimate diagnostic approach will be utilized in which information from the Structured Clinical Interview for DSM-IV [136] is supplemented by information from family informants, previous psychiatrists, and medical records to generate a diagnosis.

3. Women will need to be taking a stable dose of antipsychotic regimen for at least two months and are considered to have stable symptoms by the treating psychiatrist. This regimen must include any form of risperidone or paliperidone , or first generation antipsychotics (i.e., haloperidol, perphenazine, loxapine, or fluphenazine),

4. All women will have a prolactin level > 24 ng/ml (either identified at screening or from the past 6 weeks in the medical record).

5. All women will have evidence of a prolactin related hormonal side effect (amenorrhea, oligomenorrhea or galactorrhea) or significant sexual dysfunction. This will be determined by patient report/history and medical record/clinician interview. Oligomenorrhea is defined as infrequent, irregularly timed episodic bleeding occurring at intervals of more than 35 days from the previous menstrual cycle and amenorrhea is defined as absence of menstruation for three menstrual cycles or 6 months [137]. Galactorrhea is defined as lactation or copious milk secretion.

6. Subjects must be judged competent to participate in the informed consent process and provide voluntary informed consent, by scoring a 10 out of 12 on the Evaluation to Sign Consent (ESC).

##### Exclusion criteria

1. Postmenopausal women will be excluded. Since it may be difficult to determine menopause in patients with amenorrhea, any women more than 45 years will be assessed for menopausal symptoms, including but not limited to: hot flushes, depression, excitability and fatigue. A medical doctor will advise on the menopausal status.

2. Patients with a history of a pituitary tumor (microadenoma, macroadenoma, neoplasm) will not be included in the study. Previous medical records will be obtained if possible to examine prolactin levels and medical histories.

3. Subjects with documented Cushing’s disease, or who are pregnant or currently lactating post pregnancy will be excluded.

4. Subjects who meet DSM-IV TR criteria for alcohol or substance abuse within the last month are excluded. Subjects with nicotine use or dependence will not be excluded.

5. Medications which may affect prolactin or cause sexual dysfunction, including: metoclopramide, methyldopa, reserpine, amoxapine, droperidol, prochlorperazine, promethazine, bromocriptine, and cabergoline.

There are many medications that may affect sexual function (not hormonal side effects) unrelated to dopamine transmission. These are only permitted as long as the subject has been receiving them for greater than 4 weeks (SSRIs, mood stabilizers, diuretics, antihypertensives, H_2_antagonists, buproprion). We allow these medications to enhance generalizability.

### Study assessments

Table 1 outlines assessments performed throughout the study. In addition, a more detailed description of these assessments has been included in the text below.

**Table 1:** Schedule of events for study procedures

**Week of treatment**	**Baseline**	**1**	**2**	**4**	**6**	**8**	**10**	**12**	**14**	**16**
Physical exam, EKG, demographic information and forms	X									
Childhood Experience of Care and Abuse Questionnaire (CECA.Q), and Ways of Coping Checklist (WCC)	X									
Drug Use Information	X	X	X	X	X	X	X	X	X	X
Tobacco Craving Questionnaire, smoking questionnaire, Nicotine Dependency Form and CO (smokers only, 30 minutes post cig)	X					X				X
Fertility and Sexual Behavior Questionnaire , menstrual attitude scale	X									X
Urine pregnancy test	X	X	X	X	X	X	X	X	X	X
Vital signs, blood pressure, weight, waist circumference	X	X	X	X	X	X	X	X	X	X
Prolactin	X	X	X	X	X	X	X	X	X	X
Estradiol, progesterone*	X	X	X	X	X	X	X	X	X	X
Salivary cortisol	X					X				X
Risperidone and 9-OHrisperidone plasma levels, thyroid panel including TRH	X					X				X
CBC, Chemistry and full lipid panel (Total cholesterol, triyglycerides, HDL, LDL, VLDL), FBG, HbA1C	X					X				X
Highly sensitivity C- reactive protein (CRP)	X					X				X
Osteocalcin, bone specific alkaline phosphatase, urinary NTx and serum CTx	X			X		X				X
Cytokines	X					X				X
Parathyroid hormone, homocysteine, and 25-hydroxyvitamin D levels	X					X				X
Medication accountability and pill count		X	X	X	X	X	X	X	X	X
BPRS, SANS, CDS. CGI	X	X	X	X	X	X	X	X	X	X
RBANS	X									X
SAS, BAS, SEC	X	X	X	X	X	X	X	X	X	X
AIMS	X									X
SF-36, DAI, FSFI, FSDS-R, ASEX, PGWB	X			X		X		X		X
Menstrual diary** (kept daily) checked	X	X	X	X	X	X	X	X	X	X
Breast examination***	X		X	X	X	X	X	X	X	X
Exit Interview, Perceived Study Benefits										X

*Subjects will be given a salivary test at each visit and will be asked to perform one for the next week, refrigerate and return.

**Phone calls will be made twice weekly to remind the subjects to keep their menstrual diary daily.

***Done by female research staff or nurse (will only be repeated if galactorrhea is noted during baseline).

### Hormonal side effects

A menstrual diary is used to measure hormonal side effects, specifically her menstrual period and galactorrhea. Each woman is given a Menstrual Diary and is instructed to note any day that they experience bleeding or pain throughout the study. Study staff review the diary with participants every 2 weeks to ensure that the diary is being correctly used and to determine occurrence of menstrual bleed. This diary is a monthly calendar that asks subjects to include any day with menstrual flow (Spotting = 1, Light = 2, Moderate = 3, Heavy = 4). If a subject has galactorrhea at baseline, she is asked to note the presence of lactation daily in the diary. Participants bring the diary to biweekly assessments and are reminded to complete the diary by twice weekly phone calls. If the diary is not filled out, the subject is shown a calendar and asked to recall any menstrual bleeding in the last two weeks and weekly reminders are provided to the subject until the next visit. Regaining regular menstruation is defined as: menstrual bleeding for 3–8 days followed by another period of menstrual bleeding for 3–8 days that occurs 21–35 days after the first episode. This is the justification for the 16-week trial length. Based on pilot data the menstrual period should return within 8–12 weeks and 16 weeks should provide adequate time to see two regular menstrual periods return. Resolution of galactorrhea is defined as the absence of any lactation or milk discharge for a period of ≥ 7 days. Breast exams are done every two weeks during the study until lactation is no longer present.

### Sexual function assessments

Sexual function is evaluated using the Female Sexual Function Index (FSFI) [138] as the primary rating scale and the Arizona Sexual Experience Scale (ASEX) [139] and Female Sexual Distress Scale-Revised (FSDS-R) [140] as secondary measures. The FSFI is a brief (19-item) multidimensional scale that has been found reliable and valid for assessing sexual function in women. It has been used in women with schizophrenia and does not require subjects to have a regular sexual partner. Weigel et al. [138] found a FSFI cut point > 26.55 optimal for identifying presence of sexual dysfunction using receiver operating curves. The FSDS-R is a scale of 13 items rated 0–4 and has been found to be valid and reliable. A cutoff score >15 is used to determine presence of sexually related personal distress. The ASEX has five items rated from 1 (enhanced) to 6 (markedly impaired), with ASEX total scores ranging from 5–30. This scale has been demonstrated to be valid and reliable in people with schizophrenia or schizoaffective disorder. A total score of > 19 is indicative of sexual dysfunction. All raters for the FSFI, ASEX and FSDS-R are women to avoid any embarrassment or lack of detail in responses to male raters.

### Psychiatric symptom assessments

The following instruments are used to assess neuropsychiatric symptoms: Brief Psychiatric Rating Scale (BPRS) [141]; Scale for the Assessment of Negative Symptoms (SANS) [142]; Calgary Depression Scale (CDS) [143]; Clinical Global Impression Scale (CGI) [144] and the Repeatable Battery for the Assessment of Neuropsychological Status (RBANS) [145]. The BPRS four positive symptom items - conceptual disorganization, suspiciousness, hallucinatory behavior, and unusual thought content - are used to measure positive psychotic symptoms. The SANS total score, minus the global items, inappropriate affect, poverty of content of speech, and attention items, is used to measure negative symptoms. The inappropriate affect, poverty of content of speech, and attention items are excluded as lacking construct validity and because factor analytic study results suggest that these items are not closely related to negative symptoms [146]. The CDS total score is used to measure depressive symptoms and CGI severity of illness item is used to assess global changes. The CDS is a 9-item scale designed specifically to assess depressive symptoms in patients with schizophrenia. The CDS has been shown to be reliable and have good construct validity. Lastly, the RBANS is a brief neurocognitive battery with four alternate forms, measuring immediate and delayed memory, attention, language, and visuospatial skills.

### Subject wellness, quality of life and satisfaction with treatment

The SF-36 Health Survey (SF-36) [147]; Drug Attitude Inventory (DAI) [148]; and the Psychological General Well Being Schedule (PGWB) [149] are used. The SF-36 is a 36-item scale used to indicate general health status. It contains physical and mental concepts including behavioral functioning, perceived well-being, social and role disability and personal evaluations of health in general. It is found to be valid and reliable. The DAI is a 30-item scale that focuses on the subjective effect of antipsychotic medications in people with schizophrenia. Subjective responses are scored on a euphoric-dysphoric continuum, with scores ranging from −44 to 44. This scale has good reliability and validity. The PGWB is a 22-item scale for measuring quality of life in people with schizophrenia. It focuses on the inner personal state rather than external conditions such as income, work or home environment. It has 6 subscales: anxiety, depressed mood, positive well-being, self-control, general health, and vitality. Adherence to medication is assessed by pill count. An exit interview asks women if they received benefits and if they would like to continue treatment. The rater conducting the interview is different from anyone responsible for outcome ratings and is also blind to treatment assignment.

### Side effect measures

The Simpson-Angus Extrapyramidal Symptom Rating Scale (SAS) [150], Barnes Akathisia Scale (BAS) [151], Abnormal Involuntary Movement Scale (AIMS) and Side Effect Checklist (SEC) are used to assess side effects throughout the study.

### Laboratory assessments

Laboratory measurements are obtained including serum prolactin, cytokines, C reactive protein, lipids, fasting glucose and thyroid hormone. Estradiol, and progesterone are measured weekly by collection of a saliva sample, permitting more accurate assessment of estrogen exposure and ovulation and a less invasive or burdensome measure compared to serum or urine. Measures of bone turnover include measures of osteoblastic activity (plasma osteocalcin and bone specific alkaline phosphatase) and measures of osteoclastic activity (urinary N-telopeptide of type I collagen crosslinks (NTx) and C-telopeptide of collagen crosslinks (CTx)). Parathyroid hormone, homocysteine, and 25-hydroxy-vitamin D levels are measured at baseline, week 8 and week 16. High bone turnover is reported in schizophrenia but changes over time have not been well characterized. Elevated homocysteine levels have recently been reported to contribute to decreased bone turnover in schizophrenia. Increased osteoblastic activity indicates an increase in bone formation and increased osteoclastic activity is a measure of bone reabsorption. Normalization of prolactin levels is defined as a prolactin level < 24 ng/ml. All fasting laboratory measurements are drawn in the morning of testing prior to breakfast. Some subjects may meet eligibility criteria due to the presence of galactorrhea, but still have regular menses. To control effects of menstrual cycles on prolactin levels, menstruating subjects have randomized treatment started on the 7th day after starting their menstrual period. Cytokines are measured by Luminex Technology at the University of Maryland Core Cytokine Laboratory. The following are measured: TNF-alpha, IL-2, IL-6, IL-12, adiponectin, leptin, IFN gamma, all known to be stimulated by elevated prolactin.

### Study medication

The intervention starts at a dose of 5 mg/day of aripiprazole or matching placebo tablet and is escalated to 10 mg PO daily at the end of week 2 and further increased to 15 mg/day at the end of week 8 in women who have not yet achieved a first menstrual period. If a woman regains her menstrual period during titration, she remains on the lowest dose at which menses occurred. With this escalation plan, all women in the pilot study had first menses by 12 weeks, giving a high probability of observing two normal menstrual periods by 16 weeks. Dosing of aripiprazole or placebo may be also be modified to 5 mg/day by the clinician if side effects occur. Participants are randomized by the study statistician and treatment assignments given only to the unblinded research pharmacist. A permuted block randomization sequence is used; the randomization will be stratified by inpatient/outpatient status. During the study subjects will be maintained on the current dose of antipsychotics and antipsychotics will be dispensed utilizing the same pharmacy procedures as the double blind medication. Study physicians are instructed to avoid changing the dose of antipsychotic medication throughout the study, however this is permissible. Subjects will be able to receive anticholinergic medications as needed (e.g., benztropine and diphenhydramine) for extrapyramidal side effects, propranolol for akathisia, and benzodiazepines (e.g., lorazepam) for agitation or anxiety.

### Data analysis

All analyses will be conducted on an intention to treat basis, according to randomly assigned treatment, and will adjust for inpatient/outpatient status and baseline prolactin levels. The primary outcome of this study is the resolution of amenorrhea, oligomenorrhea, or galactorrhea, which will be analyzed at two-sided alpha = 0.05. We will compare the percentages in whom hormonal side effects have remitted (normal 3–8 day menstrual period followed by another normal 3–8 day menstrual period 21–35 days apart or resolution of galactorrhea) by end of study (week 16) in the aripiprazole versus placebo groups using a Mantel-Haenszel chi-square test at two-sided alpha = 0.05 to summarize evidence across strata defined by inpatient/outpatient status and by baseline prolactin (25–75 ng/ml and >75 ng/ml. This outcome will be potentially missing in women who withdraw before Week 16, or in women whose first normal menstrual period occurs between Weeks 12 and 16. We will assume that women who withdraw from treatment early will not achieve sustained remission of hormonal side effects. From data for women in the study, whose first normal menstrual period is before Week 12, we may estimate the probability, p_m_ that a first normal menstrual period is followed by a second normal menstrual period 21–35 days later. Then if women in each stratum have first menstrual period after Week 12, the probability that k of them have second normal menstrual period after Week 16 is given by the binomial distribution p(k) = m!/k!(m-k)! × p_m_^k^(1-p_m_ )^m-k^. For all possible outcomes for the limited number of missing cases, we may evaluate the p-value, p_mh_, of the Mantel-Haenszel chi-square test and the probability p(k), of obtaining that p-value. From this we may obtain the expected p-value, given by Σ_k_ p(k) p_mh_(k), together with 95% confidence limits on the likelihood, given the missing data pattern, of getting that p-value. These will let us see the sensitivity of our study conclusions to our assumptions about women with late first menstrual periods. Sexual functioning, general wellbeing, psychiatric symptoms, and bone turnover will be assessed using mixed models repeated measures analysis of covariance of the form: follow-up measurement = baseline measurement + treatment + week + treatment x week, where within-subject correlation of repeated measurements is assessed with an unstructured covariance model and “week” is not treated as a continuous measure but a set of categorical indicators of the week at which a measurement is collected. With this model, the “treatment” term estimates the average (across weeks) difference between treatments and the treatment x week term assesses whether this difference varies over time.

### Power for the primary outcome

We based power calculations on our preliminary data on remission of menstrual abnormalities. Shim et al. [94] found that among females who were treated with haloperidol and who had menstrual abnormalities (amenorrhea or oligomenorrhea) at baseline, 63% regained normal menstruation among those assigned to adjunctive aripiprazole and 0% for those assigned to placebo. If we assume 20% dropout by Week 16, with dropouts imputed to remain abnormal, and we assume 63% remission in completers [94], we would get 50% remission in the total sample. Based on the pilot study, we might assume a higher rate of remission with longer follow-up than seen in the 8-week study by Shim et al. Using two-sided chi-square tests at alpha = 0.05, if we assume 10% remission in women assigned to placebo, and 63% remission in completers, we would have power = 0.89 to detect this difference at with n = 25 per group; if we assume 70% remission in completers (56% in the total sample randomized), we would have power = 0.95 to detect this difference with n = 25 per group.

### Regulatory

The study has been fully IRB approved, registered in clinical trials.gov and is reviewed by a Data Safety Monitoring Board. Participants are compensated for their time.

## Discussion

Hyperprolactinemia is a common occurrence with many antipsychotics and is very often associated with distressing side effects and symptoms, particularly in women. The consequences of prolactin elevations can be profound including negative health outcomes, nonadherence and, as recently identified, suicide. We have presented details on a currently ongoing clinical trial, the DAAMSEL study, to increase recognition of prolactin-related adverse effects and treatment research around this critical issue.

## Competing interests

Dr. Kelly has received grant support from Bristol Myers Squibb for study medication only. She has served on one advisory panel for Otsuka/Lundbeck Pharmaceuticals. Dr. Buchanan served on advisory boards for Amgen, Janssen Pharmaceutica, Inc, NuPathe, Pfizer, Roche and Takeda. He is a consultant for Abbott, Amgen, Bristol Myers Squibb, EnVivo, Omeros, and Pfizer. He serves on the DSMB for Otsuka and Pfizer. Dr. McMahon serves as a statistical consultant for Amgen. All others have no conflicts or competing interests to report.

## Authors’ contributions

DLK and JCS designed the protocol and DLK oversees the study. She developed the first draft of this manuscript. All other authors assist in the clinical study and have reviewed and edited this manuscript.

## Pre-publication history

The pre-publication history for this paper can be accessed here:

http://www.biomedcentral.com/1471-244X/13/214/prepub
